# A Comparison of the Structure and Selected Mechanical Properties of Cr/Co Alloys Obtained by Casting and Selective Laser Melting

**DOI:** 10.3390/jfb15030061

**Published:** 2024-03-01

**Authors:** Leszek Klimek, Barbara Bułhak, Beata Śmielak

**Affiliations:** 1 Institute of Materials Science and Engineering, Lodz University of Technology, 90-924 Lodz, Poland; leszek.klimek@p.lodz.pl; 2 Department of Dental Techniques, Medical University of Lodz, ul. Pomorska 251, 92-231 Lodz, Poland; barbaramaria.bulhak@wp.pl; 3 Department of Prosthodontics, Medical University of Lodz, ul. Pomorska 251, 92-231 Lodz, Poland

**Keywords:** Cr/Co alloy, casting of metals, SLM technologies

## Abstract

Selective laser melting (SLM) technologies are becoming increasingly popular. The aim of the work is to compare the metallographic structure, hardness, and selected strength properties of alloys obtained by casting and by SLM, with a particular emphasis on fatigue strength. Twenty Cr/Co alloy bars were made by casting or SLM, and samples of appropriate dimensions were prepared for individual tests. The microstructures of the samples were tested by metallography, and then tested for hardness, impact strength, tensile strength, bending strength, and fatigue strength; they were also subjected to fracture after bending, tensile, fatigue, and impact tests, with the resulting fractures examined by scanning electron microscopy (SEM). Primary dendrites and small amounts of gas bubbles were present in the cast samples ground lengthwise. The SEM samples were more finer grained and uniform. Compared to the casting samples, the SLM samples demonstrated higher hardness, lower mean impact strength and higher tensile strength. The casting samples also displayed lower mean elongation values. The casting samples demonstrated slightly higher fatigue strength. The fractures of the casting samples showed an interdendritic character with clearly visible dendrites at the fracture, while those of the SLM samples were also intergranular, but finer grained. SLM generally results in better strength properties, while casting obtains slightly greater fatigue strength.

## 1. Introduction

Prosthetic restorations should be safe, resistant to the action of biochemical factors in the oral cavity and be biophysically and electrochemically neutral. In addition, they should not have a taste or smell and should be precisely shaped and durable, i.e., demonstrate acceptable mechanical properties [1,2,3]. Although recent years have seen the significant development of both new technologies and materials in Dentistry, metal-based restorations are still in use. Growing patient expectations concerning the biocompatibility, aesthetics, and durability of prosthetic restorations have driven the search for new solutions. One potential candidate is zirconium dioxide, which has been found to demonstrate high clinical effectiveness in prosthetic restorations in the anterior part of the dental arch, as well as in segmented restorations. However, due to its low tensile strength, zirconium is not suitable for complex multi-segmented prostheses. In such cases, metal base restorations are used as an alternative [4,5,6,7].

One of the oldest and most popular methods of creating metal elements in prosthetic laboratories is based on casting metal alloys using the investment casting method. Briefly, crowns and bridges with individual shapes are obtained based on wax models shaped according to the working model of the patient’s dental arch. Following this, the wax is burned and replaced with liquid metal in centrifugal or vacuum-pressure casting. However, the process requires strict procedures to be followed to ensure high quality restorations, and the dental technician is exposed to a large amount of harmful substances that may cause contact allergy, immediate type allergy, and various immune-related respiratory diseases (berylosis, cobaltosis) [8,9,10]. Moreover, casting methods result in large losses of material caused by the need to cut off the casting channels filled with metal, and the process of melting and solidification of the metals constituting the alloy causes the deterioration of the micromechanical properties of the resulting structure.

A good alternative to casting may be selective laser melting (SLM) [11,12,13,14,15,16,17]. SLM systems can also be classified as powder bed fusion (PBF), electron powder bed fusion (EPBF) or electron beam melting (EBM), selective laser sintering (SLS), and direct metal laser sintering (DMLS) [18,19,20]. The process, developed by Electro Optical Systems GmbH (EOS), allows a therapeutic structure to be created using CAD/CAM. A computer-controlled ytterbium laser beam in a chamber filled with noble gas is directed to a platform on which powdered metal is spread. The laser beam melts the material particles, which combine into a layer 20–200 μm thick. The device plate is then lowered and the sintered layer is covered with another layer of metal powder and fused again. Thus, the laser is used to fuse subsequent sections of the structure and join them together. Finally, the metal structure is thermally heated in an argon shield to eliminate stresses. Fast hardening after melting leads to the formation of a homogeneous material structure [20].

A Co-Cr-Mo alloy was first used in medical and dental technology in the 1960s. Initially, it was used as a material for denture construction. Many years of tests and research indicate that this material has excellent properties for making prosthetic frameworks and restorations: the alloy has high bending strength, high hardness, and abrasion resistance provided by cobalt, high corrosion resistance provided by chromium and molybdenum, and biocompatibility. The key advantage of this method is that it incurs virtually no material loss: any metallic powder that has not melted can be reused after sieving. The disadvantage is the cost associated with purchasing equipment and the higher cost of material. In the working chamber, an appropriate gas is selected based on the working alloy. The laser operating parameters are adjusted to minimize overheating of the material.

The obtained metal prosthetic structures should be accurate, homogeneous, and free from shrinkage cavities, empty spaces, and impurities, and should possess appropriate mechanical parameters [21,22,23,24,25,26,27,28,29]. Kiliçarslan et al. [27] confirmed that SLM is more accurate than casting: the width of the internal gap between Cr/Co alloy crowns and implant abutments ranged from 52.19 ± 11.61 μm to 140.01 ± 31.84 μm in SLM, and from 65.50 ± 9.54 μm to 313.46 ± 48.12 μm in the casting method. A literature review conducted by Koutsoukis et al. [28] indicated that Cr/Co alloy structures obtained by laser melting have minimal internal porosity, good marginal adhesion, and greater bond strength with facing ceramics compared to the casting method. The SLM structures also demonstrated better mechanical and electrochemical properties than casting. In turn, Li et al. [29] found that milled and SLM-obtained Cr/Co alloy samples showed significantly greater adhesion to porcelain than cast samples; the surface morphologies were similar, while the metallurgical structures were different.

Thanks to their numerous advantages, SLM technologies are becoming increasingly popular [20,30]. However, perhaps due to their novelty, the strength properties of Cr/Co alloys used in prosthetic restorations remain relatively poorly studied. Therefore, the aim of the present work is to compare the metallographic structure, hardness, and selected strength properties of Cr/Co alloys obtained by traditional casting and by SLM, with a particular emphasis on fatigue strength.

## 2. Materials and Methods

Twenty rods, 45 mm in length and 3 mm in diameter, were formed by casting or by SLM Co-Cr alloys. The composition of the samples was determined using an SRS 300 X-ray spectrometer (SIEMENS, Burladingen, Germany). The results are summarized in Table 1.

The specimens for the casting samples were prepared using the lost wax method. The SLM specimens were modeled and exported in an STL format. The samples were printed on an MCP Realizer (CP-HEK, Borchen, Germany). After the fusion process, the samples were annealed at a temperature of 900 °C for two hours. Samples were then taken for individual tests.

Samples for individual tests were made from pre-prepared bars.

### 2.1. Metallographic Tests

Metallographic tests were performed to compare the microstructure of samples after SLM and casting. Two metallographic sections were used for testing in the longitudinal and transverse directions relative to the rod axis. Before testing, the samples were embedded in resin, and then polished and etched in aqua regia with the following composition: three parts of hydrochloric acid (HCl) and one part of nitric acid (HNO_3_). The examination was performed on a Nikon Eclipse MA 100 microscope (Nikon Tec Corp., Tokyo, Japan). Observations were made on both longitudinal and transverse sections at magnifications of 100× and 500×.

### 2.2. Hardness Measurements

Hardness measurements were performed using the HV method (Vickers Hardnes) on the Future Tech FM device (Future-Tech Corp., Kawasaki City, Japan) based on the PN-EN ISO 6507-1 standard [31]. The load on the indenter was 10 N. Measurements were carried out on previously prepared metallographic sections. Ten measurements were performed on the cast and SLM samples.

### 2.3. Impact Tests

**Figure 1:**
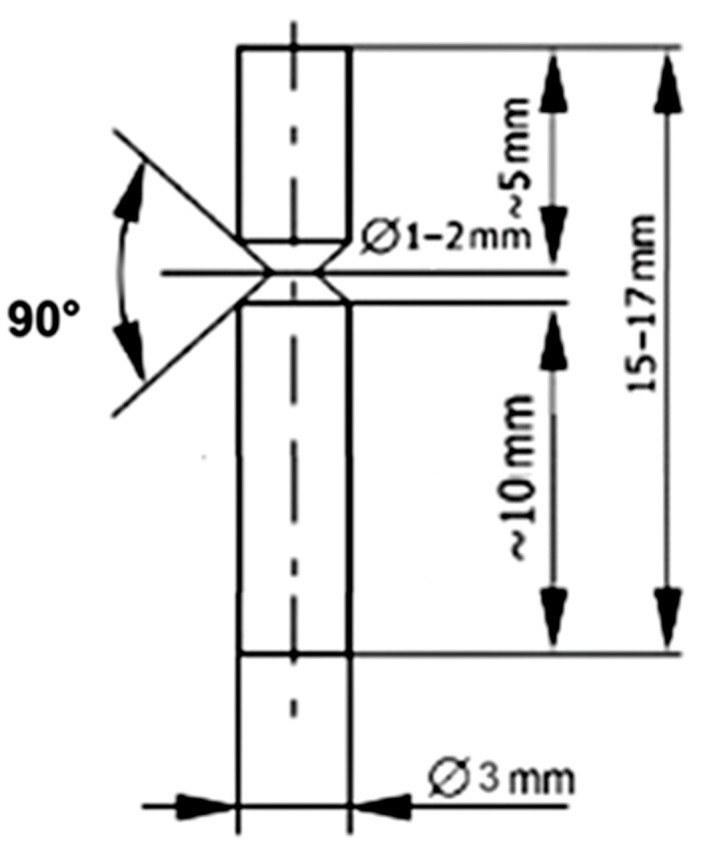
Shape and dimensions of the sample for impact strength testing.

Impact tests were carried out on the Zwick Roell HIT5.5P device (ZwickRoell GmbH & Co. KG, Ulm, Germany) based on the ISO 13802 [32]. Six samples obtained by casting and SLM were prepared for testing. The shapes and dimensions of the samples are shown in Figure 1.

Impact strength was calculated using the formula:

KC = K/S(1)

where:

KC—impact strength [J/cm^2^];

K—work needed to break the sample [J];

S—area of the broken cross-section [cm^2^].

### 2.4. Tensile Strength

**Figure 2:**
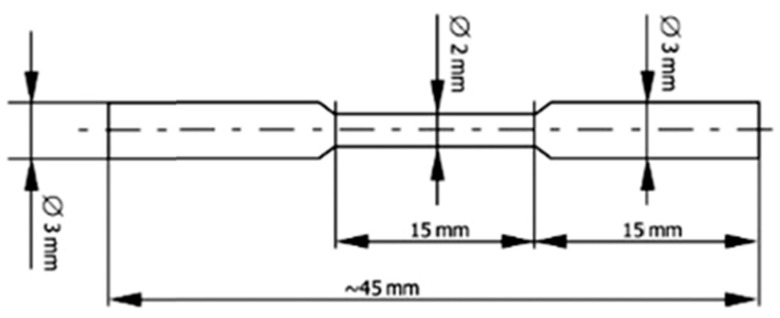
Shape and dimensions of the sample for tensile strength testing.

Tensile strength tests were carried out on a Zwick/Roell Z020 device (ZwickRoell GmbH & Co. KG, Ulm, Germany) based on the PN-EN ISO 6892-1 standard [33]. Six samples obtained by casting and SLM were prepared for testing, with dimensions given in Figure 2.

The following formula was used to calculate strength:

R_m_ = F_m_/A_0_ (2)

where:

R_m_—tensile strength [MPa};

F_m_—maximum force obtained during a tensile test [N];

A_0_—initial cross-sectional area of the sample [m^2^].

### 2.5. The Bending Test

**Figure 3:**
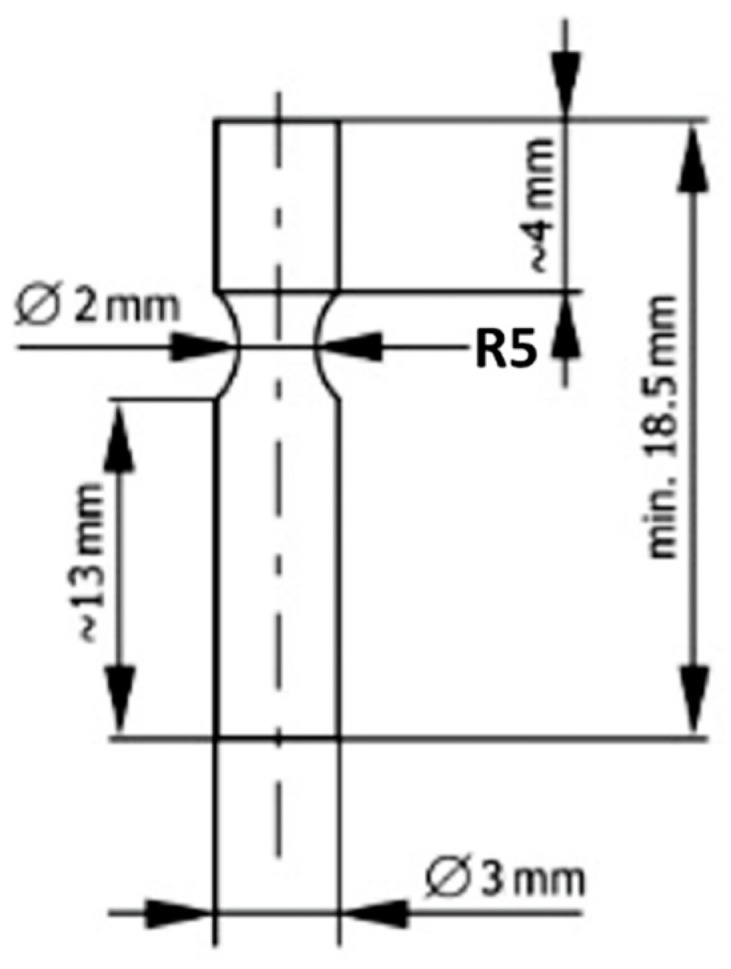
Shape and dimensions of the sample for bending strength testing.

The bending test (two-point) was performed on the Zwick Roell device (ZwickRoell GmbH & Co. KG, Ulm, Germany) based on the PN-EN ISO14955-4:2019 standard [34]. One sample obtained by casting and SLM was prepared for testing. This study was only intended to select an appropriate starting load to perform fatigue testing. The shape and dimensions of the sample are shown in Figure 3.

Bending strength was calculated from the formula:

R_g_ = M_g_/W (3)

where:

R_g_—flexural strength [kG/mm^2^];

M_g_—bending moment [kG/mm];

W—section modulus for bending [mm^3^].

### 2.6. Fatigue Strength Tests

Fatigue strength tests were performed using the two-point rotary bending method, performed on a device of our own design. Ten samples obtained by casting and fusion were prepared for testing. The shape and dimensions of the sample were the same as those for the two-point bending test (Figure 3). During the test, each sample was subjected to a different load and the number of cycles after which the sample cracked was recorded. One end of the sample was loaded with a specific force, which caused a bending moment in the narrowed part, allowing the stress in this cross-section to be calculated. The results were used to create a Wöhler curve across the limited fatigue strength range.

### 2.7. Fractographic Studies

Fractographic studies of samples were performed on the samples after completing the bending, tensile, fatigue, and impact tests. All were tested using a Hitachi S-3000N scanning electron microscope (Hitachi High-Tech, Hitachinaka, Japan) at magnifications from 30× to 500×.

### 2.8. Statistical Analysis

The results of the mechanical properties tests were subjected to statistical analysis.

Continuous variables were tested for normality using the Shapiro–Wilk test. Normally distributed variables were compared using Student’s *t*-test, and the remainder were compared with the Mann–Whitney test. *p*-values below 0.05 were considered statistically significant. For post hoc considerations regarding the minimum sample size, a significance level of α = 0.05 and power of β = 0.8 were assumed, with allocation to the compared groups in a 1:1 ratio.

## 3. Results

### 3.1. This Metallography

**Figure 4:**
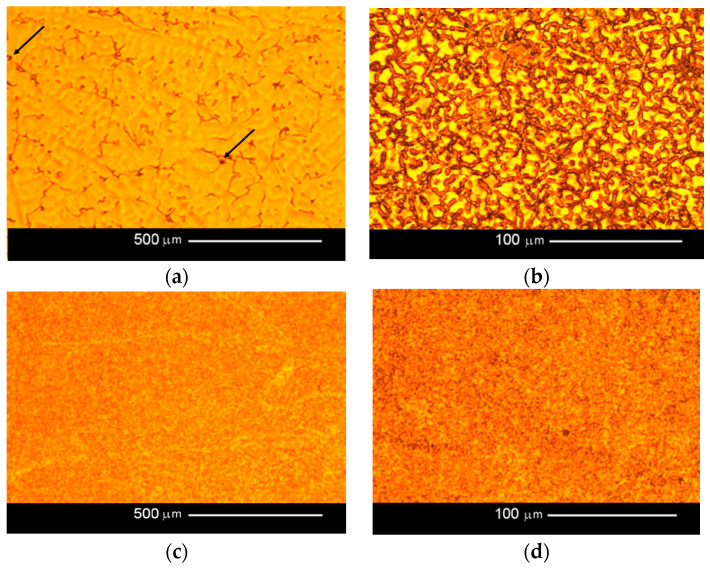
Microstructures of the tested samples: (**a**) cast sample; image plane parallel to rod axis (100× magnification); (**b**) cast sample; image plane perpendicular to rod axis (500× magnification); (**c**) SLM sample; image plane parallel to rod axis (100× magnification); (**d**) SLM sample; image plane perpendicular to rod axis (500× magnification). Example pores are marked with arrows.

The obtained microscopy images of the samples are shown in Figure 4.

In the cast samples ground lengthwise, primary dendrites can be seen, and these are particularly visible in the plane parallel to the rod axis. Small amounts of gas bubbles are also present. By comparison, the SLM samples are much more fine grained, with no primary dendrites, and the grains are rather equiaxed. Some porosity is present, but much less than in the cast samples. The structure is also more uniform after SLM.

### 3.2. Hardness

The results obtained during the Vickers hardness measurement along with the results of the statistical analysis are presented in Table 2.

It can be seen SLM results in a much higher hardness value (mean score: 579) than casting (mean score: 387). These two scores are significantly different.

### 3.3. Impact Strength

The results of the impact strength test, with their statistical analysis, are presented in Table 3.

SLM results in lower mean impact strength values than casting. However, the differences were not significant.

### 3.4. Tensile Strength

The test results obtained in the tensile test are presented in Table 4 (tensile strength) and Table 5 (elongation of the sample).

The results presented in Table 4 indicate that SLM yields significantly higher tensile strength than casting.

Casting resulted in lower mean elongation; however, the differences between the methods were not statistically significant.

### 3.5. Bending (Two-Point)

The bending strength was 944 MPa for the cast sample, and 1104 MPa for the SLM sample. These values were adopted as the value for a single cycle in fatigue tests.

### 3.6. Fatigue Tests

The fatigue strength obtained in the bending test was assumed as the strength for one cycle. Subsequent samples were loaded with different forces, which generated different stresses. The number of cycles after which the sample cracked was determined. The results showing the stress and number of cycles for individual samples are presented in Table 6.

**Figure 5:**
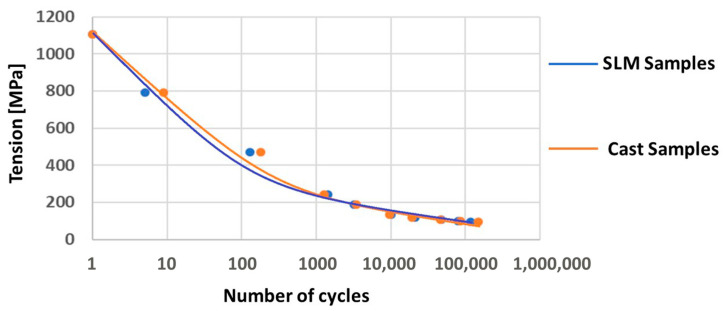
Wöhler curve for cast samples and SLM samples.

**Figure 6:**
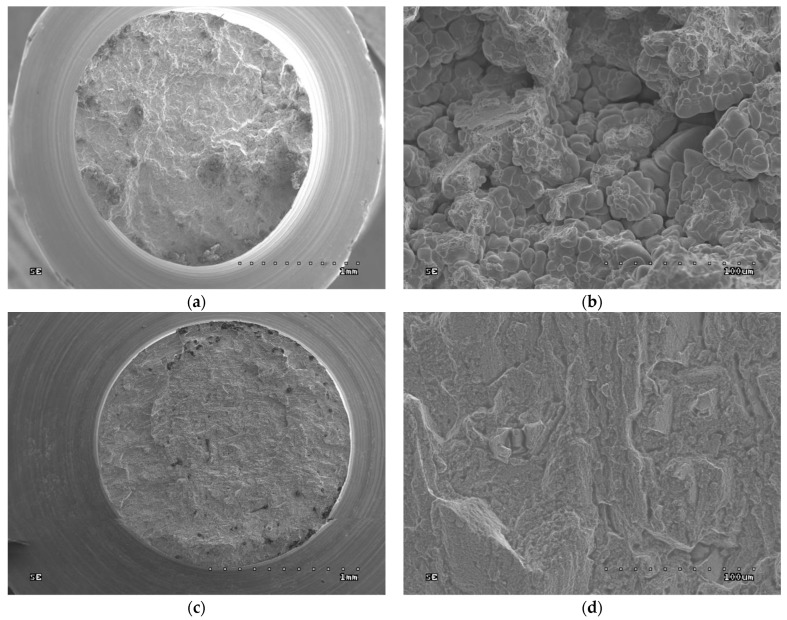
Fractures of samples after tensile tests: (**a**) cast sample, general view (30× magnification); (**b**) cast sample, enlargement of the central part of the fracture (45× magnification); (**c**) SLM sample, general view (500× magnification); (**d**) SLM sample, enlargement of the central part of the fracture (500× magnification).

The Wöhler curves based on the results are given in Figure 5 for the cast sample and Figure 6 for the SLM sample.

### 3.7. Fractographic Tests

**Figure 7:**
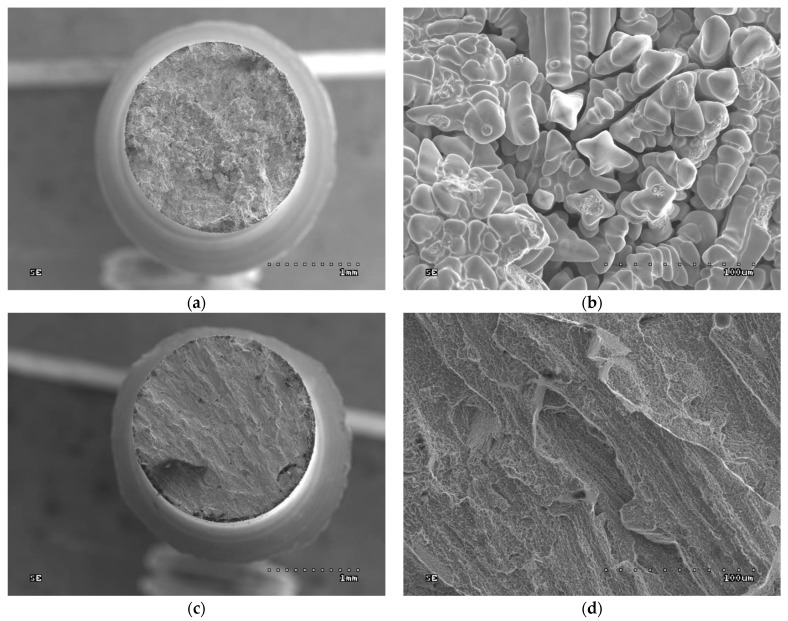
Fractures of samples after impact test: (**a**) cast sample, general view (30× magnification); (**b**) cast sample, enlargement of the central part of the fracture (500× magnification); (**c**) SLM sample, general view (30× magnification); (**d**) SLM sample, enlargement of the central part of the fracture (500× magnification).

**Figure 8:**
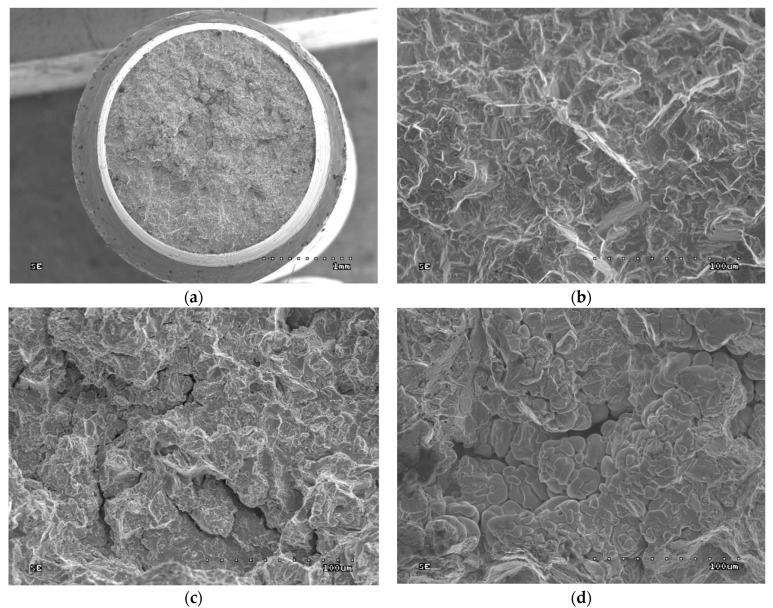
Fractures of the cast sample after fatigue tests: (**a**) cast sample, general view (500× magnification); (**b**) fatigue break zone (500× magnification); (**c**) fatigue fracture zone with fragmentation cracks (500× magnification); (**d**) residual fracture zone (500× magnification).

**Figure 9:**
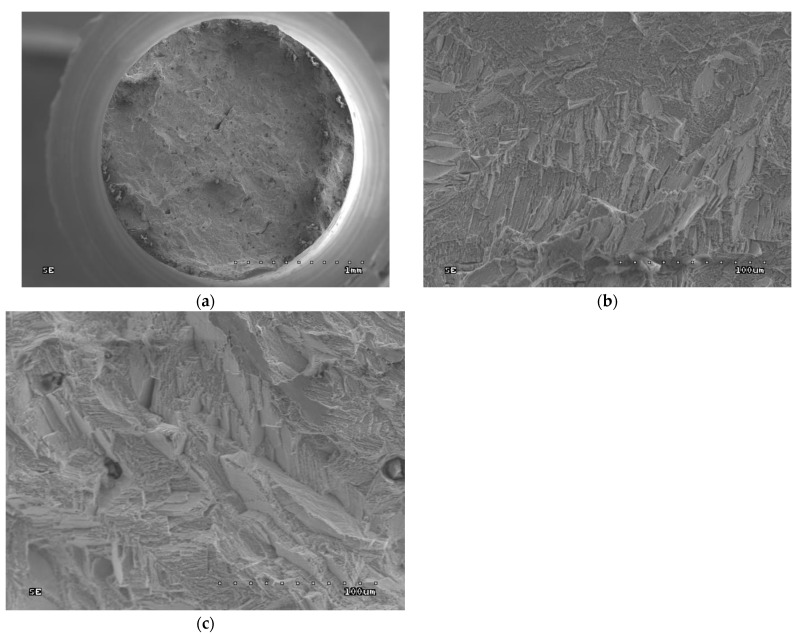
Fractures of the SLM sample after fatigue tests: (**a**) SLM sample, general view (40× magnification); (**b**) fatigue break zone (500× magnification); (**c**) residual fracture zone (500× magnification).

Fractographic tests were carried out on the fractures of the samples after strength testing. SEM images are given in Figure 6, Figure 7, Figure 8 and Figure 9.

In both the cast and SLM samples, the fractures formed by the tensile test show an intergranular character. However, the fractures of the cast samples are much coarser grained due to their different metallographic structure. As shown in Figure 4, the SLM samples have a much finer grain. In Figure 6b, the outer surfaces of the primary dendrites are clearly visible. This image indicates that this is an area of porosity where they could grow freely.

The fractures observed after the impact tests are similar to those after the tensile tests. While intergranular fractures are observed in both cases, in the cast samples, they run between the dendrites of the primary structure. Here, too, porosity can be observed in the fractures, particularly in the cast samples; this is due to the fact that these samples have larger pores, as can be seen in Figure 7.

No classic fatigue spots were observed in the fatigue fractures. This may be because cracking was initiated at the near-surface porosities, which are sites of stress concentration. No fatigue resting lines can be observed, as the samples were subjected to constant, continuous loading in the fatigue test. The fractures of the samples have an intercrystalline character.

## 4. Discussion

The microscope examinations images revealed differences between the structures of the cast and SLM samples. In the case of casting, the structure is coarsely grained with clearly visible primary dendrites; this is especially visible in Figure 4a. In the cross-section (Figure 4b), the dendritic structure is poorly defined: the observed area is perpendicular to the main axes of the dendrites. Dendrite formation results from differences in the temperature gradient near the interfaces. The crystals grow rapidly in one direction, which results in an increase in local temperature associated with the latent heat of crystallization; this in turn reduces subcooling, which prevents further crystal growth occurring in front of the crystallization front. After a break, the crystal begins to grow again in another place where there is sufficient subcooling, and this continues until the local subcooling is lost again. This process leads to the formation of tree crystals, i.e., dendrites. It is possible because there is a sufficient amount of material in the liquid phase, as is the case in casting.

In contrast, no dendrites are observed during selective melting or sintering, as both processes have very small amounts of material in the liquid phase in the heated area, and dendritic growth is not possible. The crystals “stick together” during sintering or the melting of small areas, where approximately equiaxed grains can undergo crystallization; thus, the structure is composed of small, regular grains (Figure 4c,d). While both the cast and SLM samples demonstrate material discontinuities in the form of porosity, they are more common in the cast samples. By comparison, the SLM samples are much more fine grained, with no primary dendrites, and the grains are more equiaxed. Such structural differences should result in differences in mechanical properties. It is likely that the SLM samples demonstrate greater corrosion resistance, which has been confirmed by previous studies [20,21,22,23,35,36,37,38,39,40,41].

The mean hardness of the tested samples varied significantly, ranging from 387 HV1 for the cast samples to 582 HV1 for the SLM samples. These differences in hardness can be attributed to their structures: fine-grained structures, such as those obtained by SLM, possess greater hardness than coarse-grained structures, such as the cast samples (compare Figure 4b,c). Increasing the hardness should also result in an increase in the strength properties. A high hardness value is advantageous because prosthetic frameworks must be resistant to large, complex loads under chewing conditions [18,36]. Moreover, the shape must remain stable to accommodate ceramic veneering and maintain tightness with prosthetic abutments [23,24,25,26].

The cast samples were found to have a significantly lower mean tensile strength (825 MPa) than the SLM samples (1406 MPa). These findings are consistent with those of other authors [15]. Similarly, casting also resulted in lower bending strength (944 MPa) than SLM (1104 MPa). The flexural strength results should be considered indicative only, as one sample from each process was used in the test: its purpose was to determine the initial load for fatigue tests. The better strength properties of SLM samples resulted from higher hardness and a more fine-grained structure.

In the tensile tests, the cast samples were found to have slightly lower elongation (11.0%) than the SLM samples (12.6%). This suggests that due to their finer-grained structure, the SLM samples have slightly better plastic properties. However, these differences were not statistically significant.

An important property of the material for removable prosthetic elements is their impact strength. Although the mean impact strength values for cast samples are almost twice those (2.73 J/cm^2^) of the SLM samples (1.55 J/cm^2^), they should be more resistant to cracking under dynamic loads. However, these differences were not statistically significant. As the SLM samples demonstrate greater hardness, it should be expected that they have lower impact strength; however, only preliminary conclusions should be drawn from these findings.

Prosthetic elements in the oral cavity are exposed to multiple, variable loads associated with chewing and crushing food; as such, fatigue strength is an important property of the material. However, this issue has not received much attention to date. As a prosthetic restoration in the oral cavity is typically only used for approximately five years, it is not necessary to know the fatigue strength for an unlimited time. Therefore, the test results presented in this work were performed in the range of limited fatigue strength, ending with one hundred or several dozen thousand cycles, i.e., the estimated number of chewing cycles after five years of using the denture.

The fatigue test results are presented in the form of a Wöhler fatigue curve graph, as in previous studies, in Figure 5 for the cast and SLM samples. It can be seen that the cast samples withstand a greater number of cycles for the same load, indicating slightly higher fatigue strength (Table 6). As can be seen from the graphs in Figure 5, the curves become flatter in the range of several tens of thousands of cycles; this suggests that as the number of cycles increases above this number, the stress at which the samples will crack will change only slightly.

The fatigue strength of the alloy was then estimated for the cast and SLM samples. For 150,000 cycles, the fatigue strength of the cast samples was estimated at approximately 90 MPa, compared to approximately 80 MPa for the SLM samples. It should be noted that these estimates are indicative and, in our opinion, may refer to prosthetic elements that have small cross-sections. Although strength tests relate the force to the cross-section, strength should be independent of the sample size. However, it should be borne in mind that the strength properties are determined by the structure of the alloy after the manufacturing process; this structure may vary depending on the cross-section size and method of formation, especially in the case of cast elements. Thick elements, under the same conditions, cool slower than thin ones. The choice of molding and casting technology also influences cooling, and thus the casting structure and properties.

The fractographic tests revealed differences in the fractures of samples made using different technologies. In addition, within the same group of samples, i.e., cast or SLM, no differences in fracture were observed between the fracture method, viz. impact strength, tensile strength, or bending. In the cast samples, all fractures show an interdendritic character (Figure 6a,b and Figure 7a,b) with clearly visible dendrites at the fracture. In comparison, the fractures of the SLM samples are also intergranular (Figure 6c,d and Figure 7c,d), but more fine grained. Therefore, it can be concluded that the structure of the fractures reflects the metallographic structure of the samples.

In both cases, the presence of intergranular fractures indicates reduced alloy strength at the grain boundaries. In the case of the cast samples, these may be voids around the dendrites, as evidenced by the clearly visible, undamaged dendrites in Figure 6b and Figure 7b. In the case of the SLM samples, any weakening at the grain boundaries may be caused by the accumulation of impurities there or imprecise melting. No foci that would trigger cracking were observed in the fatigue fractures. The fractures in the fatigue cracking area are not very smooth, which is due to the relatively small number of cycles. In the cast samples, local fragmentation cracks were observed on the fracture surfaces. They may be the result of the local cracks generated in the voids joining and propagating. The residual fractures found after fatigue tests are analogous to the ad hoc interdendritic fractures observed for the cast samples in the other strength tests and the intergranular fractures in the SLM samples.

To summarize, SLM processes offer better mechanical properties, in addition to high dimensional accuracy and material savings [18,19,20]. Increasing mechanical properties is of great clinical importance. Elements made using this method should be more durable, which is very important for patients undergoing prosthetic treatment. As previously mentioned, no significant differences were observed in the plastic properties (impact strength, tensile elongation) after either process, indicating that they will behave similarly. Although the estimated fatigue strength is slightly lower, the differences are so small that it should not matter when operating devices made with this technology. Therefore, throughout the entire period of use, prosthetic elements made using this method should behave similarly to elements made using the casting method. It should be noted that fatigue strength is rarely taken into account when designing and manufacturing prosthetic restoration components. This may result in unexpected damage (cracking) of these elements. Our findings indicate that the fatigue strength was less than 10% of the bending strength; therefore, long-term loading of prosthetic elements with forces much lower than the immediate strength may cause their destruction. As such, it is advisable to consider fatigue strength when designing and manufacturing prosthetic restoration elements.

## 5. Conclusions

While the samples produced by SLM treatment demonstrated better strength properties than those produced by traditional casting, they were also found to have lower fatigue strength. However, it is important to note that our fatigue test results were obtained from only a small number of samples; as such, our results should be treated as preliminary.

In addition, the course of fractures in the cracking process reflects the metallographic structure of the alloy. The mechanical properties of prosthetic elements are influenced by their manufacturing process, and this should be taken into account when designing them. In addition, as fatigue strength constitutes around 10 percent of the immediate strength, this should be taken into account when designing prosthetic elements.

## Figures and Tables

**Table 1:** Elemental composition of cast and SLM samples by weight.

	Content [% by Weight]				
Element	Cr	Si	Mo	Mn	Co
Cast alloy	28.38	2.40	3.52	1.35	rest
SLM alloy	27.87	2.87	3.29	1.25	rest

**Table 2:** Hardness measurements of cast and SLM samples.

Hardness HV1					
Type of Sample	Mean	Standard Deviation	Median	Min Value	Max Value
Cast samples	387	18	384	353	419
SLM samples	582	39	579	542	686
*p*-value	0.000034				
Distribution	Non-normal				
Result of the test	Statistically significant difference				

**Table 3:** Impact strength measurements of cast and SLM samples.

Impact strength [J/cm^2^]					
Type of Sample	Mean	Standard Deviation	Median	Min Value	Max Value
Cast samples	2.73	0.63	2.75	2.06	3.93
SLM samples	1.55	0.38	1.48	1.06	2.25
*p*-value	0.06086				
Distribution	Non-normal				
Result of the test	No statistically significant difference				

**Table 4:** Tensile test.

Tensile Strength [MPa]					
Type of Sample	Mean	Standard Deviation	Median	Min Value	Max Value
Cast samples	825	96	806	730	983
SLM samples	1406	77	1406	1291	1468
*p*-value	0.000779				
Distribution	Normal				
Result of the test	Statistically significant difference				

**Table 5:** Elongation measurements of cast and SLM samples.

Elongation [%]					
Type of Sample	Mean	Standard Deviation	Median	Min Value	Max Value
Cast samples	11.0	1.5	10.6	9.7	13.1
SLM samples	12.6	1.8	12.0	10.0	14.6
*p*-value	0.288				
Distribution	Normal				
Result of the test	No statistically significant difference				

**Table 6:** Fatigue strength test results.

Sample No	Cast Samples		SLM Samples	
	Tension	Number of Cycles Failure	Tension MPa	Number of Cycles Failure
1	1107	1	1107	1
2	792	9	792	5
3	471	178	471	180
4	244	1270	244	1443
5	189	3422	189	3200
6	136	9611	136	10.001
7	117	11.395	117	11.327
8	106	46.705	106	47.126
9	100	85.496	100	81.117
10	94	148.613	94	118.164

## Data Availability

Data are contained within the article.
