# Correction: Klimek et al. A Comparison of the Structure and Selected Mechanical Properties of Cr/Co Alloys Obtained by Casting and Selective Laser Melting. *J. Funct. Biomater.* 2024, *15*, 61

**DOI:** 10.3390/jfb15060159

**Published:** 2024-06-06

**Authors:** Leszek Klimek, Barbara Bułhak, Beata Śmielak

**Affiliations:** 1 Institute of Materials Science and Engineering, Lodz University of Technology, 90-924 Lodz, Poland; leszek.klimek@p.lodz.pl; 2 Department of Dental Techniques, Medical University of Lodz, ul. Pomorska 251, 92-231 Lodz, Poland; barbaramaria.bulhak@wp.pl; 3 Department of Prosthodontics, Medical University of Lodz, ul. Pomorska 251, 92-231 Lodz, Poland

In the original publication [1], there was a mistake in Figure 4 as published. Three sub-pictures (Figure 4b–d) were the same in Figure 4. The corrected Figure 4 appears below. The authors state that the scientific conclusions are unaffected. This correction was approved by the Academic Editor. The original publication has also been updated.

## Figures and Tables

**Figure 4:**
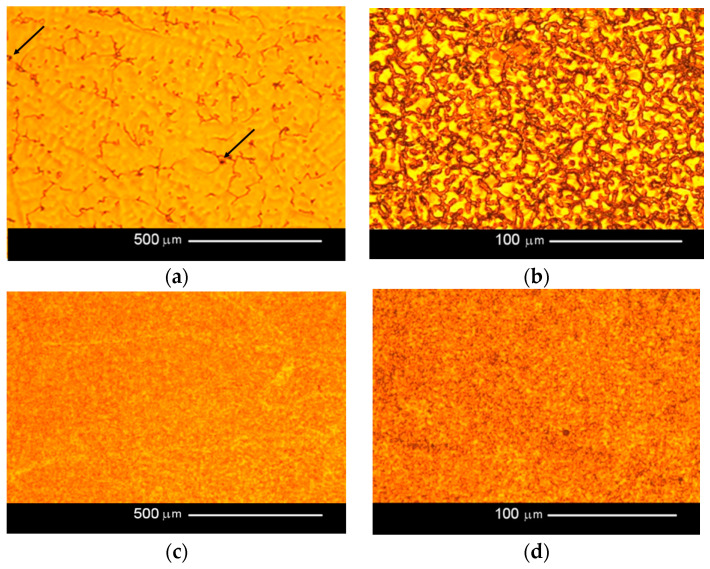
Microstructures of the tested samples: (**a**) cast sample; image plane parallel to rod axis (100× magnification); (**b**) cast sample; image plane perpendicular to rod axis (500× magnification); (**c**) SLM sample; image plane parallel to rod axis (100× magnification); (**d**) SLM sample; image plane perpendicular to rod axis (500× magnification). Example pores are marked with arrows.
